# Renal Autotransplantation for Resection of Bilateral Nephroblastoma and High-Risk Neuroblastoma in Children

**DOI:** 10.3390/cancers17060989

**Published:** 2025-03-15

**Authors:** Benjamin F. B. Mayer, Matthias C. Schunn, Cristian Urla, Lea Weinpert, Ilias Tsiflikas, Martin Ebinger, Frank Fideler, Felix Neunhoeffer, Marcus Weitz, Silvio Nadalin, Steven W. Warmann, Jörg Fuchs

**Affiliations:** 1 Department of Paediatric Surgery and Paediatric Urology, University Children’s Hospital Tübingen, Hoppe-Seyler Straße 3, 72076 Tübingen, Germany; cristian.urla@med.uni-tuebingen.de (C.U.); lea.weinpert@stud.uni-tuebingen.de (L.W.); joerg.fuchs@med.uni-tuebingen.de (J.F.); 2 Department of Paediatric Surgery, Charité University Hospital Berlin, Augustenburger Platz 1, 13353 Berlin, Germany; matthias.schunn@charite.de (M.C.S.); steven.warmann@charite.de (S.W.W.); 3 Division of Paediatric Radiology, Department of Diagnostic Radiology, University Hospital Tübingen, Hoppe-Seyler Straße 3, 72076 Tübingen, Germany; ilias.tsiflikas@med.uni-tuebingen.de; 4 Department of Paediatric Hematology, Oncology, Gastroenterology, Nephrology and Rheumatology, University Children’s Hospital Tübingen, Hoppe-Seyler Straße 1, 72076 Tübingen, Germany; martin.ebinger@med.uni-tuebingen.de (M.E.); marcus.weitz@med.uni.tuebingen.de (M.W.); 5 Department of Anesthesiology and Intensive Care Medicine, University Hospital Tübingen, Hoppe-Seyler Straße 3, 72076 Tübingen, Germany; frank.fideler@med.uni-tuebingen.de; 6 Department of Paediatric Cardiology, Pulmonology and Intensive Care Medicine, University Children’s Hospital Tübingen, Hoppe-Seyler Straße 3, 72076 Tübingen, Germany; felix.neunhoeffer@med.uni-tuebingen.de; 7 Department of General, Visceral and Transplant Surgery, University Hospital Tübingen, Hoppe-Seyler Straße 3, 72076 Tübingen, Germany; silvio.nadalin@med.uni-tuebingen.de

**Keywords:** bilateral wilms tumor, bilateral nephroblastoma, high-risk neuroblastoma, renal autotransplantation, partial nephrectomy

## Abstract

Background/Objectives: In bilateral nephroblastoma and high-risk neuroblastoma in children with extensive tumor involvement of the renal vessels or pedicle, complete tumor resection with preservation of healthy renal tissue is not feasible with in situ nephron-sparing surgery or vascular replacement. The aim of this study was to present our experience with ante situ tumor resection and renal autotransplantation (RATX) in these children. Methods: A retrospective study of children with bilateral nephroblastoma and high-risk neuroblastoma who underwent tumor resection and RATX at an international referral center for pediatric surgical oncology between 2006 and 2024 was performed. RATX was performed by transection of renal vessels, ante situ mobilization, and perfusion of the kidney with Bretschneider’s solution. Tumor resection was performed on a bloodless kidney under hypothermia. Results: Ante situ tumor resection and RATX were performed at a median age of 36 months (range 13–62) in 4 children with bilateral nephroblastoma and 4 children with high-risk neuroblastoma. Complete tumor resection was achieved in all patients. One patient with neuroblastoma died of sepsis after 14 days. The 7 surviving patients showed no evidence of disease and normal to slightly decreased glomerulofiltration rates at a median follow-up of 20 months (range 3–155). Limitations include the retrospective design, small sample size, and heterogeneity of the study population due to very rare indication. Conclusions: Ante situ tumor resection and RATX is a feasible surgical approach for children with multifocal bilateral nephroblastoma or high-risk neuroblastoma who are ineligible for in situ nephron-sparing surgery or vascular reconstruction.

## Simple Summary

For children with large tumors in or around the kidneys, such as bilateral nephroblastoma or high-risk neuroblastoma, surgery to remove the tumors and spare healthy kidney tissue is often not possible. This article describes an alternative surgical technique: The kidney is moved outside the body, cooled on ice, and fed with a solution. The tumor can then be removed from the kidney outside of the body, and the kidney can then be reimplanted into the body. We have found that with this method, tumors can be completely removed while saving healthy kidney tissue.

## 1. Introduction

Nephroblastoma is the most common malignant renal tumor to manifest in childhood, with approximately 5% to 10% being bilateral, while neuroblastoma is the most common malignant abdominal tumor with encasement of the renal pedicle [1]. For both tumor entities, complete tumor resection is the primary surgical goal [2,3]. However, long-term effects of treatment must be considered when planning surgical resection. Due to underlying germline genetic aberrations, end-stage renal disease is the most clinically significant morbidity in patients with bilateral nephroblastoma [4]. Renal dysfunction is the most common long-term sequela in neuroblastoma following nephrotoxic systemic treatment [5]. To achieve complete tumor resection while preserving healthy renal tissue, in situ nephron-sparing surgery (NSS) has become the gold standard for surgical resection of bilateral nephroblastoma [6,7,8]. To avoid renal damage, the ischemia time should be as short as possible and should not exceed 30 min to preserve renal function [9,10]. Therefore, this approach has limitations in cases of extensive multifocal tumor involvement, with the infiltration of the renal hilus and collecting system. In resection of high-risk neuroblastoma, the nephrectomy rate, which can be as high as 9%, can be reduced by preoperative identification of renal vascular abnormalities and partial nephrectomy in the case of renal tumor infiltration [11,12,13]. In cases of vascular injury to the aorta or renal vessels, vascular reconstruction by suture or patch application may not be possible and nephrectomy may be required [14].

In these distinct conditions of bilateral nephroblastoma and high-risk neuroblastoma, complete tumor resection with preservation of healthy renal tissue can be achieved by performing ante situ tumor resection and renal autotransplantation (RATX). For this purpose, the renal vessels are transected and hypothermia and perfusion of the kidney with histidine–tryptophan–ketoglutarate (HTK) solution is established. For bilateral nephroblastoma, tumor resection can then be performed ante situ, i.e., outside the abdominal cavity, leaving the ureter intact using the same technique as in situ NSS. For neuroblastoma, the intraabdominal tumor resection can be completed, and the kidney can be reimplanted orthotopically or in the iliac fossa at the end of the surgery. To date, this procedure has been described in a few cases for resection of bilateral nephroblastoma at a time or institution where in situ NSS was not established [15,16,17]. For resection of high-risk neuroblastoma, one case has recently been reported in Russian language [18]. Due to the paucity of the literature on this surgical technique, the aim of this study was to describe our experience with ante situ tumor resection and RATX for bilateral nephroblastoma and high-risk neuroblastoma.

## 2. Materials and Methods

### 2.1. Study Design

A retrospective single-center study of children with bilateral nephroblastoma and high-risk neuroblastoma who underwent ante situ tumor resection and RATX between 2006 and 2024 was performed. All patients were treated according to the protocols of the German and International Societies for Pediatric Oncology and Hematology, including neoadjuvant chemotherapy [19,20]. Patients with bilateral nephroblastoma underwent preoperative mercaptoacetyltriglycine (MAG) scintigraphy or magnetic resonance urography (MRU) to assess renal split function [21]. Due to the inherent risk of 50% renal failure, patients with Wilms tumor–aniridia–genitourinary anomalies–mental retardation (WAGR), Denys–Drash, and other syndromes associated with Wilms tumor protein 1 (WT1) mutations were not considered for ante situ tumor resection with RATX [4]. National and local multidisciplinary tumor boards indicated for ante situ tumor resection and RATX for multifocal bilateral nephroblastoma with tumor involvement and infiltration of the renal hilus and collecting system in cases where R0 tumor resection and renal pelvic reconstruction by NSS with in situ vascular exclusion could not be performed within 30 minutes. Indication for RATX in resection of neuroblastoma was made intraoperatively in cases of tumor infiltration or injury of renal vessels where vascular repair or replacement was not possible. All surgeries were performed by the senior author and RATX in collaboration with a transplant surgeon. Data were retrieved from hospital records and stored in a computerized database (Microsoft Excel, Version 1808, Microsoft Corporation, Redmond, WA, USA).

### 2.2. Ante Situ Tumor Resection and Orthotopic Renal Autotransplantation for Bilateral Nephroblastoma

**Figure 1:**
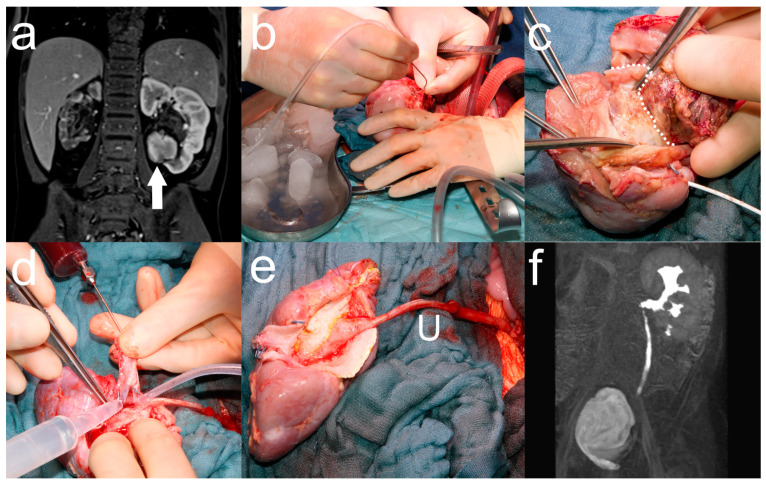
Ante situ tumor resection and orthotopic renal autotransplantation for bilateral nephroblastoma. A 3-year-old boy with bilateral nephroblastoma and dysplastic right kidney and central tumor of the left lower pole (arrow) on preoperative MRI (**a**). Ante situ mobilization of the left kidney on sterile ice water and rinsing with HTK solution (**b**). Resection of the central parts of the tumor (above the white dotted line) (**c**). Blood drawn from the patient being injected into the renal vessels to identify and suture vascular injuries (**d**). Reconstructed left kidney with ureter (U) after tumor resection (**e**). Maximum intensity projection (MIP) of postoperative MR urography showing adequate function of the reconstructed left kidney (**f**).

Tumor resection was performed in a modified fashion based on the approach used for in situ NSS at our institution (Figure 1) [22]. A transverse upper abdominal laparotomy was performed, and the kidney was completely mobilized by dissecting all the surrounding tissues. The renal vessels were freed at their origin and dissected far into the periphery to visualize the segmental branches. Intraoperative contrast-enhanced sonography was performed by intravenous injection of 0.5 mL of INN–sulfur hexafluoride (SonoVue, BRACCO Imaging Deutschland GmbH, Konstanz, BW, Germany) followed by 5 mL of saline chaser. All localized tumor foci were marked on the renal surface by electrocautery. Prior to vascular exclusion, systemic heparin (100 IU/kg) was administered. The renal artery was clamped with a Yasargil clamp to avoid intimal injury, and the renal vein was clamped with a bulldog clamp. The renal artery and vein were sharply dissected, leaving a vessel stump of at least 5 mm in length. The kidney was then mobilized anteriorly to the abdominal wall to avoid tumor intraabdominal tumor spillage, thereby leaving the ureter intact. The kidney was immediately placed in sterile ice water and intermittently flushed with HTK solution through the renal artery (Bretschneider, Custodiol^®^, Dr. Franz Köhler Chemie GmbH, Bensheim, HE, Germany) to avoid renal ischemia. After incision of the renal capsule, the shortest route through the renal parenchyma was chosen, and the parenchyma was separated from the tumor by blunt and sharp dissection. In the case of renal pelvic involvement, care was taken to leave a sufficient margin of tumor-free renal pelvic tissue for subsequent reconstruction of the collecting system. Fresh frozen sections were obtained from all resection margins to verify R0 resection. After tumor resection, hemostasis was achieved by suturing the central vessels and electrocoagulating the peripheral vessels. Blood was collected from the patient and injected into the renal vessels to identify and suture vascular injuries. The kidney was then flushed again with HTK solution to prevent thrombus formation. The collecting system was reconstructed and checked for leaks. A double-J catheter (Optimed Medizinische Instrumente GmbH, Ettlingen, BW, Germany) was placed in cases of extensive reconstruction of the collecting system. The kidney was reconstructed with mattress sutures, sealed with human fibrinogen (TISSEEL^®^, Baxter International, Deerfield, IL, USA) and thrombin (TachoSil^®^, Corza Medical, Westwood, MA, USA), and reimplanted orthotopically. The renal artery and vein anastomosis was performed end-to-end with a continuous absorbable monofilament 7-0 suture (PDS II, Ethicon Inc., Raritan, NJ, USA) using magnifying loops (5×). Before reperfusion, ice water was replaced with warm water to achieve renal vasodilation, and the anesthesiologist maintained mean arterial blood pressure above 60 mmHg. Venous reperfusion was followed by arterial reperfusion. A second intraoperative ultrasound was performed to verify complete tumor resection and restored renal perfusion. Lymph node sampling was performed according to the Renal Tumor Study Group protocol and included at least six paraaortic, parahilar, and paracaval lymph nodes [3]. A pararenal drain and a urethral catheter were left in place to detect leakage and monitor urine production. Postoperative follow-up was performed in the pediatric intensive care unit (PICU), with special care to maintain mean arterial blood pressure above 60 mmHg. Periodic ultrasound controls were performed to assess renal perfusion and to rule out urine leakage.

### 2.3. Renal Autotransplantation in Neuroblastoma Resection

**Figure 2:**
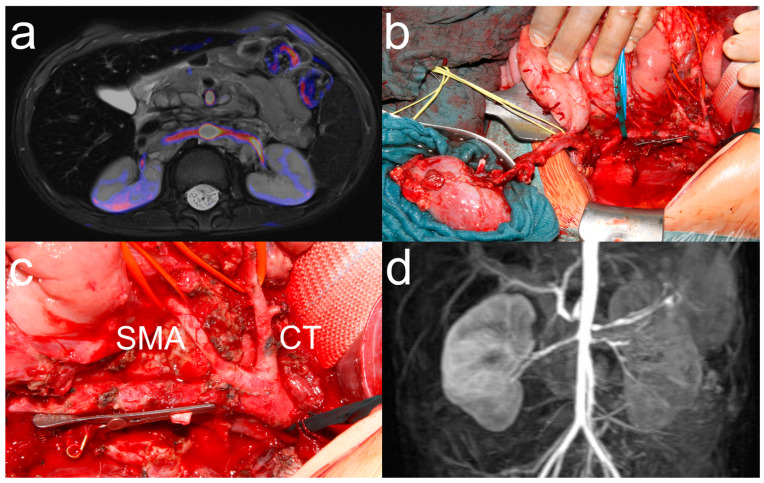
Renal autotransplantation for resection of high-risk neuroblastoma. A 5-year-old girl with high-risk neuroblastoma involving both renal arteries on preoperative image fusion of transversal T2-weighted sequence and MR angiography (**a**). Intraoperatively, the left renal vein had to be resected due to complete tumor infiltration. The kidney was mobilized ante situ (**b**) with the ureter left in place (yellow loop, blue loop: inferior vena cava). Aorta (**c**) with Yasargil clamp on the stump of the renal artery. The superior mesenteric artery (SMA) and coeliac trunk (CT) are slung with red vessel loops. Early postoperative angio-MRI showing slight irregularity of the left renal artery on MIP and consequently weaker contrast enhancement of the left kidney after renal autotransplantation (**d**).

For RATX in neuroblastoma resection (Figure 2), the renal artery and vein were dissected, leaving as many tumor-free vessel segments as possible for reanastomosis. If the vessel length was sufficient, RATX was performed orthotopically; otherwise, RATX was performed in the iliac fossa. In cases of vascular injury to the aorta at the site of the renal arteries where there was massive bleeding and immediate reconstruction was required, ante situ organ management was performed simultaneously by a second surgical team. Ante situ organ management, vascular anastomosis, and postoperative care were performed as described in the previous section.

### 2.4. Demographic and Clinical Characteristics

Demographic characteristics included age and sex. Preoperative clinical characteristics included tumor entity, risk group, tumor genetics, history of previous surgery, neoadjuvant radiotherapy, type of preoperative imaging (MRI/CT/MRU/MAG scintigraphy/ultrasound) and glomerulofiltration rate (GFR) at surgery.

### 2.5. Study Outcome

The primary outcomes of interest were operative time, duration of mechanical ventilation, length of PICU and hospital stay, postoperative complications, resection status (R0 resection/gross total resection/subtotal resection), GFR, hypertension treatment, and oncologic outcome (complete remission/local control/progression/death) at follow-up. Postoperative complications were classified according to the Dindo–Clavien classification [23].

### 2.6. Statistical Analysis

Patients who underwent ante situ tumor resection and RATX were analyzed using descriptive statistics (Microsoft Excel, Microsoft Corporation, Redmond, WA, USA). GFR was calculated using the cKiD Under 25 formula [24].

## 3. Results

Between 2006 and 2024, a total of 65 tumor resections were performed for bilateral nephroblastic lesions. Ante situ tumor resection and RATX were performed in 4 children with bilateral nephroblastoma (Table 1). Hypertension was diagnosed preoperatively in all children. Preoperative MAG scintigraphy showed complete loss of function of the contralateral kidney in 1 patient. This patient underwent ante situ resection and RATX and nephrectomy of the contralateral kidney as a single-stage procedure (Table 2, Figure 1). In the other 3 children, nephrectomy or NSS of the contralateral kidney was performed as a staged procedure after adequate renal function of the kidney with RATX was confirmed by MAG scintigraphy. R0 resection was achieved in all children. Secondary placement of a double-J catheter was required in 2 patients due to urinary leakage. At a median follow-up of 11 months (range 3–17) there was no evidence of disease (NED) in all children. All children remained on antihypertensive medication.

**Figure 3:**
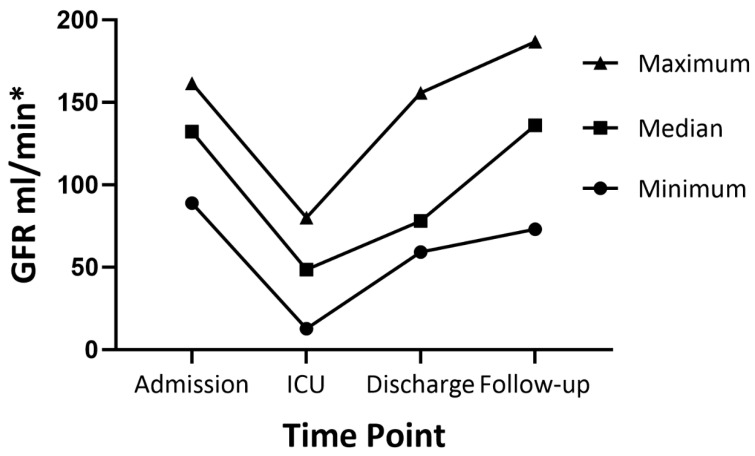
Glomerulofiltration rate in patients undergoing ante situ tumor resection with renal autotransplantation. * Glomerulofiltration rate was calculated using the cKiD Under 25 formula [22].

Of the 188 children who underwent resection for neuroblastoma with renal involvement, repair of vascular injury to the aorta and renal vessel was not possible in 4 children and RATX was performed (Table 1). Tumor resection had been attempted previously in 2 patients and 1 patient had received neoadjuvant radiotherapy. Indication for RATX was tumor infiltration of the renal vein in 1 patient and intraoperative injury of the aorta with massive bleeding in 3 patients. Gross total resection of more than 98% of visible tumors was achieved in all children (Table 2). RATX was performed orthotopically in 2 patients and in the iliac fossa in 2 other patients. Major postoperative complications occurred in 3 children. One patient developed sepsis after discharge to his home country and required short-term hemodialysis. This patient underwent secondary nephrectomy of the kidney without RATX due to complete loss of renal function. After 5 months, one patient developed bowel obstruction, requiring bowel resection. One patient developed sepsis due to chronic Klebsiella pneumonia infection and died in the PICU 14 days after surgery. At a median follow-up of 20 months (range 3–155), there was NED in the 3 surviving patients. Preoperatively, GFR was normal in all patients with values > 90 mL/min (Figure 3). Postoperatively, GFR decreased to less than 80 mL/min in all patients, but increased throughout discharge and follow-up period, with only 2 patients having mild renal insufficiency with GFR values of 51.2 and 73 mL/min.

## 4. Discussion

In our study, R0 tumor resection and preservation of renal function were possible with ante situ tumor resection and RATX for bilateral nephroblastoma ineligible for in situ NSS. With this approach, bilateral nephrectomy, dialysis and tumor spillage with subsequent upstaging to local stage III, and local irradiation were avoided in all patients [20]. Another advantage of ante situ resection was that the ureter could be left intact, in contrast to the previously proposed complete ex vivo tumor resection with transection and anastomosis of the ureter [15,16,17]. Postoperative replacement or placement of DJ catheter was required in 2 patients due to persistent urine leakage (Figure 1 c,e). However surgical complications could be managed well, and all patients recovered rapidly from surgery, allowing early initiation of adjuvant chemotherapy within 3 weeks. Hypertension persisted after surgery in all patients, but no renal artery stenosis was observed. However, the follow-up and time since the last chemotherapy session were short and the hypertension could still be reversed, as a study by Maas et al. showed that 91% of patients with nephroblastoma and hypertension had normalized blood pressure after surgery [27].

The concept of ante situ tumor resection and RATX for resection of bilateral nephroblastoma in children was first described by Longaker et al. in 1990 in one patient [17] and by Desai et al. in 1999 in three patients [15], at a time when the standard of care for all children with bilateral nephroblastoma was bilateral nephrectomy, hemodialysis, and renal transplantation [20]. As in situ NSS became the standard surgical approach for tumor resection of bilateral nephroblastoma [6,7,8,28], the surgical approach was not reported until Zhong et al. recently published a series of 8 children who underwent ex vivo tumor resection and orthotopic RATX for bilateral nephroblastoma [9]. In situ NSS with vascular exclusion for resection of these tumors was not considered by the authors of this study. The reported outcome was death in 1 patient with Denys–Drash syndrome and bilateral nephrectomy with allogeneic kidney transplantation in another patient due to renal failure.

Due to the complexity of the procedure and the high risk of renal failure, we would recommend ante situ tumor resection and orthotopic RATX as a last nephron-sparing approach when R0 resection is clearly not possible with in situ NSS within an ischemia time of 30 min [22,29]. Using this surgical strategy, tumor resection of bilateral nephroblastic lesions was successfully performed in 65 cases at our institution between 2006 and 2024, and could even be performed as a redo NSS in 9 patients experiencing local relapse [30]. Ante situ resection and orthotopic RATX were required only in the 4 children reported in this study, and bilateral nephrectomy was required only in 1 patient with Denys–Drash syndrome. Our conclusion is that in patients with bilateral nephroblastoma, rigorous patient selection with centralized review by reference surgeons, oncologists, and radiologists is critical in selecting the most appropriate surgical approach to achieve R0 resection and renal preservation with the least risk of surgical complications.

For patients with high-risk neuroblastoma and intraoperatively, irreparably compromised renal perfusion, RATX allowed for complete or gross total tumor resection with kidney preservation in our study. Three patients with prior radiation or surgery required RATX and nephrectomy of the contralateral kidney due to intraoperative vascular injury. While surgeons should always be prepared to manage vascular injury in patients with prior local treatment, we believe that the possibility of RATX should also always be considered before performing nephrectomy [14]. In our patient cohort, out of 188 patients who underwent resection for neuroblastoma with renal involvement, 9 patients underwent nephrectomy and 4 patients underwent RATX. The resulting nephrectomy rate of 5% was comparatively lower than the SIOPEN results of 9% [11]. This is particularly important because the standard treatment protocol for high-risk neuroblastoma consists of high-dose chemotherapy, immunotherapy, stem cell transplantation, and radiation therapy, all of which are highly nephrotoxic. Therefore, our recommendation for surgical resection of high-risk neuroblastoma with renal involvement is to perform advanced preoperative imaging with combined MRI and contrast-enhanced CT to identify renal vascular abnormalities, partial nephrectomy for tumor infiltration of renal tissue, and RATX for renal vascular injury or tumor infiltration [12,13].

The results of this study should be interpreted within its limitations. The main limitation is the retrospective design, small sample size, and heterogeneity of the study population, with different tumor entities and indications for RATX. This introduces significant selection, performance, and attrition bias. Since bilateral nephroblastoma and high-risk neuroblastoma are rare, and the number of patients eligible for ante situ tumor resection and RATX is even smaller, international multicenter analyses are needed for further evaluation.

## 5. Conclusions

Ante situ tumor resection with RATX is a highly complex surgical procedure that is feasible in children with bilateral nephroblastoma who are ineligible for in situ NSS. In children with neuroblastoma and intraoperatively compromised renal perfusion that cannot be restored by vascular repair or replacement, RATX allows for preservation of the kidney. Given the complexity of the procedure, we strongly recommend that this technique be performed only as a last-resort nephron-sparing approach by surgeons experienced in NSS.

## Figures and Tables

**Table 1:** Demographic and clinical characteristics.

ID	Tumor Entity	Age Dx [mon]	Sex	Disease Stage *	Tumor Genetics	Risk Group *	Preoperative Imaging	Renal function [Left:Right]	Age TR [mon]
1	BN	36	M	V	-	Intermediate	MRI + CT + MAG	98:2	41
2	BN	17	F	V	-	Intermediate	MRI + MAG	55:45	20
3	BN	8	F	V	-	Intermediate	MRI + CT + MAG	47:53	13
4	BN	58	M	V	-	Intermediate	CT + MRU	17:83	62
5	HN	23	M	III	N-MYC+	High-risk	MRI + CT	-	40
6	HN	55	F	IV	-	High-risk	MRI	-	62
7	HN	22	F	IV	N-MYC+	High-risk	MRI + CT	-	29
8	HN	12	F	IV	N-MYC+	High-risk	MRI + CT	-	32

Age Dx: Age at diagnosis, mon months; BN: bilateral nephroblastoma; HN: high-risk neuroblastoma; MRI: magnetic resonance imaging; CT: computed tomography; MAG: renal mercaptoacetyltriglycine scintigraphy; MRU: magnetic resonance imaging urography; Age TR: age at tumor resection. * Staging and classification according to the Renal Tumor Study Group of the International Society of International Paediatric Oncology (SIOP-RTSG) and the International Neuroblastoma Risk Group Staging System [25,26].

**Table 2:** Study Outcome.

ID	Operative Time [min]	Ischemia Time [min]	Contralat. Nx	Resection Status	Ventilation [d]	LPICU [d]	LOHS [d]	Complications *	Follow-Up [m]	Oncologic Outcome
1	389	120	yes	R0	2	3	14	I	27	CR
2	423	90	yes	R0	2	2	36	IIIb	17	CR
3	314	120	no	R0	2	3	21	I	7	CR
4	444	110	yes	R0	0	1	27	IIIb	3	CR
5	590	-	secondary	GTR	2	7	88	IVa	155	CR
6	450	240	no	GTR	2	7	18	IIIb	20	LC
7	585	-	yes	GTR	14	14	14	V **	-	Death
8	659	-	no	GTR	4	7	19	I	3	LC

Min: minutes; Contralat. Nx: Contralateral nephrectomy; R0: microscopic complete resection; GTR: gross total resection ≥ 98% of visible tumor; d: days; LPICU: length of stay at pediatric intensive care unit; LOHS: Length of hospital stay; m: months; CR: complete remission, LC: local control. * Classification according to Dindo-Clavien [21]. ** Patient died of Sepsis in PICU.

## Data Availability

All data generated or analyzed during this study are included in this article or available upon request. Further inquiries can be directed to the corresponding author.

## Abbreviations

The following abbreviations are used in this manuscript:

CT	Computed tomography
GFR	Glomerulofiltration rate
HTK	Histidine–tryptophan-–ketoglutarate
MAG	Mercaptoace–tyltriglycine
MIP	Maximum intensity projection
MRU	Magnetic resonance urography
NED	No evidence of disease
NSS	Nephron-sparing surgery
PICU	Pediatric intensive care unit
RATX	Renal autotransplantation
WAGR	Wilms tumor–aniridia–genitourinary abnormalities–mental retardation syndrome
WT1	Wilms tumor protein 1
